# Recent Advances in the Development of Mammalian Geranylgeranyl Diphosphate Synthase Inhibitors

**DOI:** 10.3390/molecules22060886

**Published:** 2017-05-27

**Authors:** Staci L. Haney, Veronica S. Wills, David F. Wiemer, Sarah A. Holstein

**Affiliations:** 1 Department of Internal Medicine, University of Nebraska Medical Center, Omaha, NE 68198, USA; staci.haney@unmc.edu; 2 Department of Chemistry, University of Iowa, Iowa City, IA 52242, USA; vwills87@gmail.com (V.S.W.); david-wiemer@uiowa.edu (D.F.W.)

**Keywords:** geranylgeranyl diphosphate synthase, isoprenoid, geranylgeranylation, inhibitors

## Abstract

The enzyme geranylgeranyl diphosphate synthase (GGDPS) catalyzes the synthesis of the 20-carbon isoprenoid geranylgeranyl diphosphate (GGPP). GGPP is the isoprenoid donor for protein geranylgeranylation reactions catalyzed by the enzymes geranylgeranyl transferase (GGTase) I and II. Inhibitors of GGDPS result in diminution of protein geranylgeranylation through depletion of cellular GGPP levels, and there has been interest in GGDPS inhibitors as potential anti-cancer agents. Here we discuss recent advances in the development of GGDPS inhibitors, including insights gained by structure-function relationships, and review the preclinical data that support the continued development of this novel class of drugs.

## 1. Introduction

**Figure 1:**
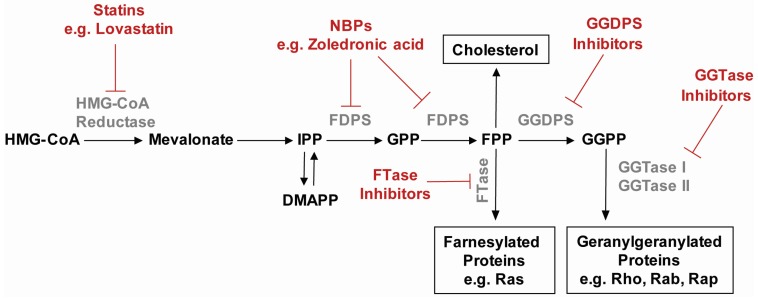
The mammalian isoprenoid biosynthetic pathway with associated inhibitors.

The isoprenoid biosynthetic pathway (IBP) is one of the most heavily targeted biochemical pathways in medicine, with millions of individuals currently taking statins to lower cholesterol levels or nitrogenous bisphosphonates to address bone disease. Entry into the mammalian IBP begins when HMG-CoA reductase (HMGR) converts 3-hydroxy-3-methylglutaryl-coenzyme A (HMG-CoA) to mevalonate via the rate-limiting step in the pathway (Figure 1). Mevalonate is phosphorylated and then decarboxylated to yield isopentenyl pyrophosphate (IPP), which can reversibly isomerize to dimethylallyl pyrophosphate (DMAPP). Both DMAPP and IPP serve as substrates for farnesyl diphosphate synthase (FDPS) which generates first the 10-carbon geranyl pyrophosphate (GPP) and then the 15-carbon farnesyl pyrophosphate (FPP). In a reaction mediated by the enzyme geranylgeranyl diphosphate synthase (GGDPS), FPP and IPP are condensed to yield the 20-carbon compound geranylgeranyl diphosphate (GGPP).

**Figure 2:**
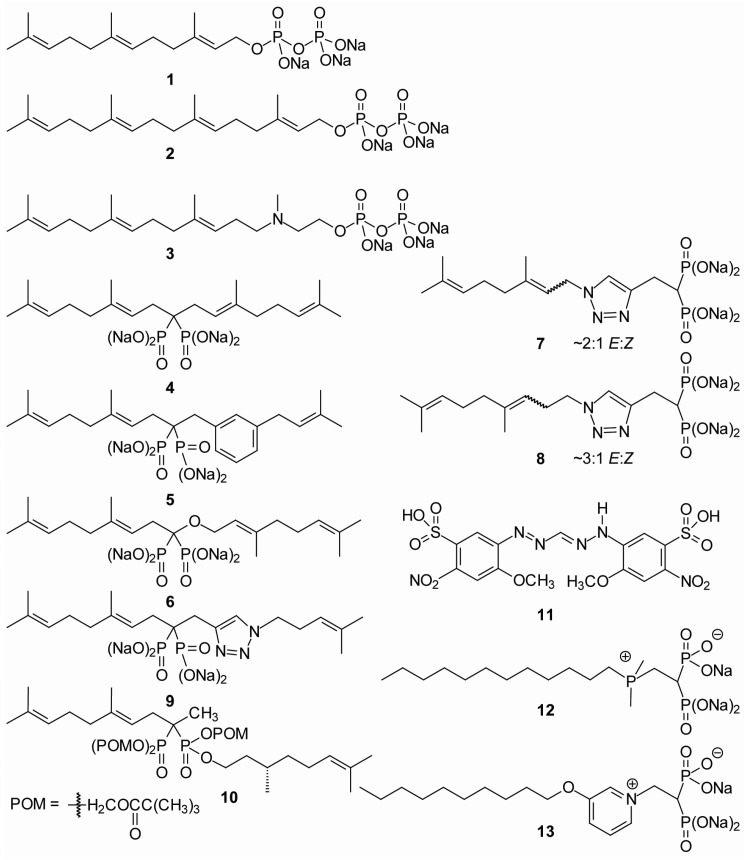
Chemical structures of select geranylgeranyl diphosphate synthase (GGDPS) inhibitors.

Two key products of the IBP, FPP and GGPP (**1** and **2**, Figure 2), serve as substrates for farnesyl transferase (FTase) and geranylgeranyl transferases (GGTase I and II), respectively. These enzymes play a critical role in the posttranslational modification of the Ras small GTPase superfamily of proteins (e.g., Ras, Rab, and Rho families). Prenylation refers to the addition of a 15-carbon isoprenoid chain (via farnesylation) or a 20-carbon isoprenoid chain (via geranylgeranylation) onto a carboxy terminal cysteine residue of a protein. Rab and Rho proteins are geranylgeranylated, whereas Ras proteins are typically farnesylated. Substrates of FTase and GGTase I share a consensus C-terminal sequence (the “CAAX” box) which dictates enzyme recognition. However, GGTase II is unable to recognize Rab proteins directly and instead utilizes the Rab escort protein (REP) which delivers Rab to the enzyme’s active site and allows prenylation to occur [1]. 

The Ras family of proteins, including H-, K-, and N-Ras, play critical roles in regulating cellular proliferation in normal and cancer cells. The Rho family of proteins is primarily involved in cytoskeletal reorganization, but also plays a role in the survival of malignant cells. Rab proteins regulate nearly all aspects of intracellular membrane trafficking processes, including facilitating vesicle budding, motility, docking, and fusion. Prenylation of the Ras superfamily members, including the Rab proteins, is essential to ensure proper cellular localization and function. For example, mutant Rab proteins, that cannot be geranylgeranylated, are mislocalized and nonfunctional [2]. Given the diverse roles of prenylated proteins in cellular functions, there has been extensive interest in the development of agents which disrupt protein prenylation by inhibiting the IBP. In this review, we discuss IBP inhibitors with particular focus on GGDPS inhibitor development.

## 2. Statins and Nitrogenous Bisphosphonates

The most widely used IBP-inhibiting drugs are statins for the treatment of hypercholesterolemia. Statins inhibit HMG-CoA reductase, which is well-recognized as the first committed step in isoprenoid biosynthesis. While statins do inhibit endogenous cholesterol biosynthesis, their cholesterol-lowering effects are secondary to increased clearance of LDL from the plasma due to upregulation of the hepatic LDL receptor [3,4]. There is also evidence that statins influence cardiovascular health via other mechanisms, including putative effects on vascular inflammation, endothelial function and myocardial remodeling [5]. There has been considerable interest in the use of statins in other clinical indications, including cancer, neurological disorders, osteoporosis, asthma, coagulation, and thrombosis [6,7,8,9,10,11]. The anti-cancer activities of statins appear related to their ability to disrupt protein prenylation [12]. The use of statins as anti-cancer agents in vivo, however, is likely to be limited. In vitro studies have demonstrated that the concentrations of statins required to affect prenylation are in the low micromolar range, while the concentrations needed to lower cholesterol biosynthesis are in the nanomolar range [13]. Standard dosing regimens result in serum drug levels of ~0.1 μM [14], thus it is likely that only cholesterol synthesis is impacted. Several phase I studies involving oncology patients have demonstrated that administration of high-dose statin can yield serum drug levels in the low micromolar range, however the higher doses were associated with side effects [15,16]. These findings fueled investigation into the development of agents that inhibit downstream events in the IBP pathway as a means to inhibit protein prenylation more directly.

Nitrogenous bisphosphates (NBPs) represent the second class of clinically used agents that target the IBP. Bisphosphonates can be viewed as non-hydrolysable analogues of pyrophosphate and their phosphorus-carbon-phosphorus backbone facilitates chelation of calcium ions. This results in a high affinity for bone, which ultimately leads to a high concentration in osteoclasts. In the clinic, NBPs are prescribed extensively for the treatment of osteoporosis and metastatic bone disease because they inhibit bone resorption. Interestingly, these agents were used clinically prior to an understanding of the molecular target. The first clue that NBPs may inhibit the IBP pathway came in the late 1990s when it was noted that NBPs disrupt protein prenylation [17,18]. Further investigation revealed the enzyme FDPS as the target of NBPs [19,20]. Inhibition of FDPS by NBPs depletes cells of both FPP and GGPP, thus disrupting all types of protein prenylation within a cell. NBPs effects on prenylation have been observed in a wide range of cell types, including macrophages, osteoclasts, osteoblasts, and endothelial cells, as well as various cancer cell lines. While anti-cancer activity has been observed in vitro, the benefit in vivo is likely substantially less given the limited systemic distribution of these drugs [21,22,23]. Despite this limitation, results from clinical trials suggest that the NBPs may provide therapeutic advantages that extend beyond skeletal-related events [21]. For instance, zoledronic acid is a potent FDPS inhibitor with an IC_50_ of 3 nM [24]. A study in breast cancer patients found that zoledronic acid inhibited cellular proliferation and induced apoptosis when used as a monotherapy [25] and there is evidence in myeloma that zoledronic acid has treatment benefits beyond improving bone health [26].

## 3. GGDPS Inhibitors

In 1992, Sagami et al., reported 3-aza-2,3-dihydrogeranylgeranyl diphosphate (3-azaGGPP) (**3**, Figure 2) as an inhibitor of GGDPS [27]. Since it was modelled upon GGPP, 3-azaGGPP contains a longer carbon chain than the previously developed bisphosphonates and specifically inhibited GGDPS, but not FDPS, isolated from rat liver. In 1999, Kuzuguchi et al. reported the cloning, expression and partial characterization of human GGDPS [28]. While FDPS and GGDPS share limited sequence homology, the five amino acid motif characteristic of trans-prenyl transferases is present in both enzymes. This fueled investigation into bisphosphonates and other compounds resembling FDPS inhibitors as potential inhibitors of GGDPS. Overall, the differences in the structures between the two synthases have allowed for the development of distinct sets of specific inhibitors. While the most potent of the FDPS inhibitors are small molecules containing a nitrogen heterocycle and a bisphosphonate head group, the majority of the GGDPS inhibitors prepared to date (described below) incorporate one or more longer alkyl chains into the bisphosphonate-based structure.

**Figure 3:**
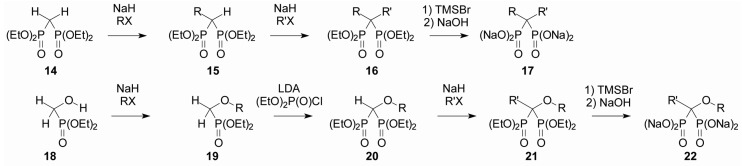
Synthetic sequences to dialkyl (**17**) and alkyl/alkoxy (**22**) bisphosphonates.

In 2002, a set of bisphosphonates and azaprenyl diphosphates was reported with IC_50_ values against purified GGDPS enzyme ranging from 0.14–690 μM [29]. Such results suggested that bisphosphonate compounds containing isoprenoid chains might also function as inhibitors of GGDPS. These prior studies prompted the design of a library of isoprenoid bisphosphonates, including digeranyl bisphosphonate (DGBP, **4**, Figure 2), which inhibited geranylgeranylation of small GTPases, but not farnesylation [30]. Shortly thereafter, it was discovered that DGBP specifically inhibits GGDPS, resulting in depletion of intracellular GGPP [31]. DGBP was reported to have an IC_50_ value of ~200 nM against purified enzyme and inhibited protein geranylgeranylation, but not farnesylation, in cultured cells [31]. Structure-function work suggested that with regard to the alkyl substituents on the carbon central to the phosphonates, at least one geranyl chain (or a longer isoprenoid) was required for significant inhibition of GGDPS. Crystallographic studies showed that DGBP’s V-shaped structure occupied the enzyme’s active site, with the bisphosphonate group complexing with magnesium ions and the two prenyl side chains occupying the substrate-binding site of FPP, as well as the product-binding site of GGDPS [32]. Preparation of the isoprenoid bisphosphonates proved to be relatively straightforward (Figure 3). When treated with base and an alkyl halide, commercial tetraethyl methylene bisphosphonate (**14**) can be alkylated to obtain the monoalkyl derivative **15**, and the process can be repeated to introduce a second alkyl substituent (**16**). Final hydrolysis readily affords the corresponding salts **17**. Evaluation of a series of mono- and dialkylated compounds prepared in this way revealed that the di-substituted compounds are generally more potent than the mono-substituted compounds [33]. Assessment of a series of aromatic bisphosphonates (e.g., **5**, Figure 2) demonstrated selective inhibition of GGDPS over FDPS, albeit at lower potency than DGBP [34]. Through a similar strategy, a series of geminal bisphosphonate ethers was prepared based upon the hypothesis that incorporation of the ether moiety would lower the pKa3 and improve activity. For this series, the commercial phosphonate **18** was first converted to an ether through a classical Williamson synthesis. After introduction of the ether, treatment of phosphonate **19** with base and diethyl chlorophosphate resulted in C-P bond formation and gave the bisphosphonate **20**. Subsequent C-alkylation (to **21**) and hydrolysis of the phosphonate esters give the desired ethers **22**. The most potent reported example of this set, the *O,C*-digeranyl geminal bisphosphonate **6** had an IC_50_ of 82 nM against GGDPS in an in vitro enzyme assay [35].

**Figure 4:**
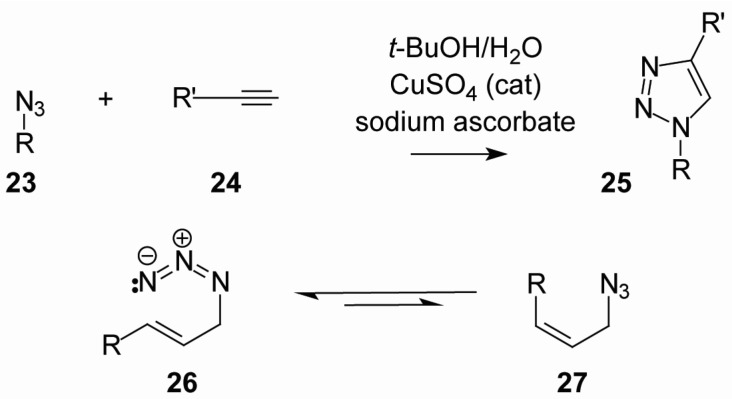
A convergent synthesis to triazole bisphosphonates.

More recently, a novel series of GGDPS inhibitors containing a triazole moiety has been reported (Table 1). The synthesis of these triazoles takes advantage of an azide-acetylene cycloaddition or “click reaction” [42]. As shown in Figure 4, click chemistry involves the cycloaddition of an azide (**23**) with a terminal acetylene (**24**), resulting in an efficient synthesis of the 1,2,3-triazole (**25**). As both the acetylene and the azide components can carry substituents, this allows for a combinatorial approach to drug development. The process is highly reliable and quite efficient for preparation of triazoles with simple alkyl or aryl substituents. However, with allylic azides, the reaction can be more complicated because of a rapid sigmatropic rearrangement which gives a mixture of *E*- and *Z*-olefins (e.g., **26** and **27**) [43]. Thus, the first triazole examples bearing geranyl or neryl substituents derived from the azide component gave a mixture of olefin isomers in a 2:1 ratio irrespective of the olefin stereochemistry in the initial azide.

**Figure 5:**
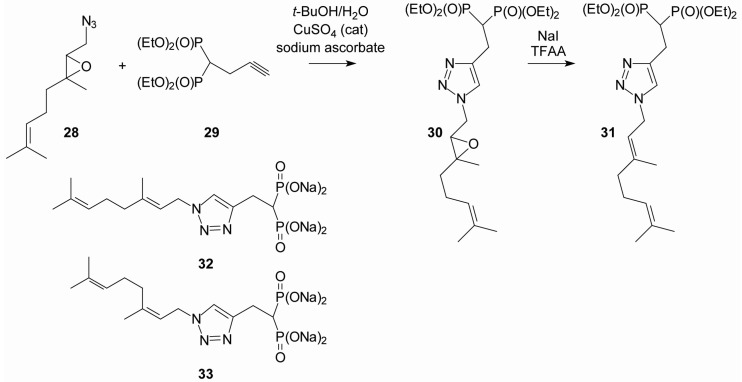
Stereoselective synthesis of isoprenoid triazole bisphosphonates through the use of epoxy azides [39].

Upon bioassay it was noted that a 2:1 mixture of the geranyl and neryl triazole bisphosphonates (**7**) globally disrupted cellular geranylgeranylation, even though it showed no activity as an inhibitor of GGTase II [39,44]. To circumvent the olefin isomerization and prepare the individual isomers, it has proven possible to mask the olefins as an epoxide (e.g., **28**), conduct the cycloaddition with the acetylene bisphosphonate **29** [42] to obtain the triazole **30**, and then reduce the epoxide to regenerate the original olefin stereochemistry (**31**) (Figure 5) [39]. This approach was utilized to generate the individual geranyl (**32**) and neryl (**33**) isomers. When the two compounds were tested in vitro against purified enzymes, the neryl isomer **33** was found to be approximately 40-fold more potent than the geranyl isomer **32** (IC_50_ 375 nM versus 17 μM), suggesting that the olefin stereochemistry has a significant impact on inhibitory activity against GGDPS [39]. The neryl isomer was also 10-fold more potent than the geranyl isomer in cellular assays, highly specific for GGDPS, and showed no activity against any of the prenyltransferases [39]. 

Additional structure-function work led to the development of homoisoprenoid triazole bisphosphonates [37]. In all tested cases, the elongation of the alkyl chain length by one-carbon enhanced activity of the compound as a GGDPS inhibitor (Table 1). The most potent of this homologated series of compounds was a mixture of homogeranyl and homoneryl triazole bisphosphonates (**8**, Figure 2, Table 1). This mixture inhibits GGDPS with an IC_50_ value of 45 nM, the lowest IC_50_ value reported to date [37]. Furthermore, the mixture was highly specific for GGDPS and displayed potent inhibition of protein geranylgeranylation in myeloma cells at concentrations as low as 30 nM [37,40]. Subsequent preparation of the individual isomers allowed for comparisons between the two isomers and revealed that the homoneryl isomer is more potent than the homogeranyl [40,45] (Table 1). Interestingly, however, there is evidence that the two isomers interact in a synergistic manner to inhibit GGDPS [40]. Computer modeling suggests that both isomers can bind simultaneously to GGDPS, with the homoneryl isomer binding preferentially at the FPP site and the homogeranyl isomer preferring the GGPP site of the enzyme [40]. Further studies are needed to explain why the synergistic interaction between the two olefin isomers is maximized when the two isomers are in a 3:1 homogeranyl:homoneryl ratio. This observation may be related to their individual affinity for the enzyme or to the oligomeric nature of GGDPS. Prior crystallography studies suggested that GGDPS assumes a hexameric organization composed of three dimers [46]. Regardless of the mechanism underlying the synergy, the homogeranyl/homoneryl triazoles represent a new paradigm in the development of GGDPS inhibitors.

Further extension of the chain to a bishomogeranyl length abrogated activity, suggesting that the homogeranyl length is the optimal length for inhibitory activity in this motif [38] (Table 1). The observed activity of the bishomogeranyl/neryl series further confirmed the importance of olefin stereochemistry as the bishomoneryl triazole was noted to be more potent than the corresponding bishomogeranyl [38]. Finally, longer-chained derivatives, including farnesyl, homofarnesyl, geranylgeranyl, and homogeranylgeranyl, have weak or no activity as GGDPS inhibitors (Table 1) [36,41]. A V-shaped dialkylated triazole bisphosphonate (**9**) containing homoprenyl and geranyl substituents did display inhibitory activity (IC_50_ 0.38 μM) (Figure 2), but was less active than the mono-alkylated homogeranyl triazole [37].

While generation of more potent inhibitors remains a worthwhile pursuit, with highly charged compounds efforts to improve a drug’s ability to cross the cell membranes are also critical. Preparation of a prodrug, which carries little or no charge, enables compounds to transit biological membranes, and then releases the parent drug once inside the target cell [47]. Preparation and testing of a series of pivaloyloxymethyl (POM)-modified mono- and dialkyl bisphosphonates revealed that the addition of the POM groups as protecting groups significantly increased the hydrophobicity and improved potency in a cellular geranylgeranylation assay by as much as 25-fold [48]. A second series of POM-prodrugs was reported by Foust et al., that incorporated one isoprenoid chain on the α-carbon, a second alkyl chain as a phosphonate ester, and the potential for a third alkyl chain at the α-carbon [49]. Among the five bisphosphonates tested, the methyl derivative (**10**, Figure 2) was the most potent, with activity noted at 0.5 μM in cell assays. 

While the majority of published GGDPS inhibitors contain the bisphosphonate backbone, non-bisphosphonate inhibitors also have been described (**11**, Figure 2) [50]. These inhibitors had IC_50_ values of ~30–50 μM and consistently bound to the FPP or GGPP site of GGDPS. Despite their lower potency, the development of non-bisphosphonate compounds may still be of interest, as these compounds could have different peripheral distribution relative to bisphosphonate-based inhibitors. Finally, zwitterionic compounds based on phosphonium (**12**) and pyridinium (**13**) motifs have been reported that have dual activity against FDPS and GGDPS [32,51]. Collectively, development of a diverse group of GGDPS inhibitors has occurred and efforts to refine the delivery and potency of these agents are currently underway.

## 4. Cellular Effects of GGDPS Inhibition

Several of the GGDPS inhibitors described above have effects on cellular proliferation and cell death in vitro [34,37,49,52]. The mechanism by which this occurs is not always clear, although one would reason that it is caused by a disruption of protein geranylgeranylation [53]. While Rho and Rab proteins predominately function as regulators of cytoskeletal reorganization and vesicle trafficking, respectively, some reports suggest they play a role in cellular proliferation and survival as well. RhoA has been implicated in several studies. Apoptosis induced by IBP inhibitors can be rescued by either exogenous delivery of GGPP or expression of constitutively active RhoA [54,55,56]. In breast cancer cell lines, expression of Rab27B promotes cell cycle progression, proliferation, and invasion and this activity is dependent on the geranylgeranylation of Rab27B [57]. In mesothelioma cells, inhibition of Rab6 geranylgeranylation activates the apoptotic pathway [58]. In addition to their role in regulating cell proliferation, GGDPS inhibitors also influence autophagy. In both breast cancer and prostate cancer cell lines, Wasko et al. demonstrated that inhibition of FDPS and GGDPS, but not FTase or GGTase I promotes autophagy as measured by accumulation of the autophagy marker LC3-II [59]. Studies performed in myeloma cells utilizing lovastatin, DGBP, or specific GGTase I or II inhibitors demonstrated that IBP inhibition induces a net increase in autophagy as a consequence of disruption of isoprenoid biosynthesis which is not recapitulated by direct geranylgeranyl transferase inhibition [60].

Given the role of geranylgeranylated Rab proteins in regulating intracellular vesicle trafficking and secretion, one would predict that inhibition of Rab geranylgeranylation would affect cellular secretion. In fact, inhibitors of the IBP pathway have been shown to disrupt normal cellular secretion in numerous cell types. Lovastatin was reported to decrease secretion of cholecystokinin in endocrine cells [61]. Another statin, simvastatin, reduced IgM secretion in lymphoplasmacytic lymphoma cell lines [62]. Our group demonstrated that disruption of Rab geranylgeranylation, either through depletion of GGPP or by inhibition of GGTase II, inhibits the secretion of monoclonal protein in multiple myeloma cells [52]. The novel triazole-based GGDPS inhibitors potently disrupt monoclonal protein secretion [37,40]. The monoclonal protein subsequently accumulates within the endoplasmic reticulum, resulting in induction of the unfolded protein response (UPR) pathway and ultimately apoptosis in multiple myeloma cells [52]. During times of reversible stress, the UPR slows protein synthesis, upregulates chaperone proteins and promotes degradation of misfolded protein. However, in instances of prolonged stress, such as continuous inhibition of protein trafficking as caused by Rab inhibitors, the UPR can activate apoptotic pathways. Multiple myeloma cells express near maximal levels of UPR-associated proteins to aid in the secretion of continuously synthesized proteins. It is for this reason that they are particularly sensitive to induction of the pro-apoptotic arm of the UPR [63]. Thus, GGDPS inhibitors may be particularly relevant with respect to anti-myeloma therapy and represent a novel strategy by which to induce myeloma cell death.

## 5. In Vivo Studies Using GGDPS Inhibitors

While limited in scope, studies performed in mice suggest that GGDPS inhibitors are active in vivo, and thus, may prove beneficial in the treatment of cancer and other diseases. In a mouse model of prostate cancer, a DGBP analog was found to disrupt Rap1a geranylgeranylation and decrease adrenal gland tumor burden by ~50% [64]. A follow-up study assessed the ability of the inhibitor to slow the development of prostate cancer metastasis in a preventative murine model [65]. Treatment of mice with this inhibitor resulted in a reduction of whole body tumor burden and prolonged survival compared to vehicle-treated animals. A pyridinium bisphosphonate (**12**) proposed to function as a dual FDPS-GGDPS inhibitor delayed tumor growth in SK-ES-1 sarcoma cells in a mouse xenograft model [51]. Collectively, these studies suggest that GGDPS inhibitors may have anti-tumor activity in vivo. 

In addition to their application as anti-cancer drugs, GGDPS inhibitors may be of use for the treatment of fibrotic lung disease [66]. The small GTP-binding protein, Rac1, mediates hydrogen peroxide (H_2_O_2_) production in alveolar macrophages and H_2_O_2_ is directly linked to the development of fibrosis. It was hypothesized that because geranylgeranylation regulates Rac1 activity, GGDPS inhibitors may render Rac1 inactive resulting in a decrease in H_2_O_2_ production. In fact, treatment with DGBP inhibited Rac1 import into the mitochondria, leading to impaired oxidative stress and an attenuated fibrotic response [66]. 

## 6. GGDPS Expression

Several studies have suggested that there is an association between GGDPS expression and disease pathology. Yu et al. examined GGDPS expression in hepatocellular carcinoma specimens. Compared with specimens that were within 2 cm or 5 cm from the tumor margin, levels of GGDPS (mRNA and protein) were higher in the tumor tissues [67]. These studies also suggested a correlation between the GGDPS protein level and the presence of cirrhosis, tumor stage, and early recurrence. GGDPS has been identified as a target gene of early growth response 1 (EGR-1) [68]. In a mouse model of cigarette smoke exposure, GGDPS and EGR-1 levels were increased in lung epithelial cells and knockdown of GGDPS resulted in a decrease in the inflammatory response induced by cigarette smoke exposure [68]. This group subsequently postulated that increased expression of GGDPS results in enhanced geranylgeranylation of Ras proteins, which in turn, leads to activation of the mitogen-activated protein kinase (MAPK) pathway [69]. Similar findings were reported in a model of insulin resistance in which hyperinsulinism induced MAPK activity in adipocytes in an EGR-1-dependent manner. Knockdown of GGDPS led to increased insulin sensitivity [70]. Thus, these studies suggest that pursuit of GGDPS inhibition as a therapeutic strategy may be of interest in disease settings characterized by GGDPS overexpression. 

## 7. Conclusions

Over the last decade, a diverse group of bisphosphonate and non-bisphosphonate based GGDPS inhibitors has been synthesized. The more recently developed isoprenoid triazole bisphosphonates display high potency and strong preference for GGDPS over other IBP pathway enzymes. Pre-clinical studies suggest that GGDPS inhibitors have promise as novel cancer therapeutics, and there may be reason to pursue their development for non-malignant disease processes as well. Significant work remains to render compounds suitable for clinical trials. One of the biggest hurdles to surpass may be drug delivery. As the majority of GGDPS inhibitors currently synthesized contain highly charged bisphosphonate backbones, their systemic distribution and entry into cells may be compromised. The use of a prodrug form may mitigate the problem, and efforts are currently underway to develop such compounds. Future studies also should focus on the toxicology and efficacy of these compounds in mouse models.

## Figures and Tables

**Table 1:** Summary of alkyl-substituted triazole bisphosphonates.

Alkyl Group	GGDPS IC_50_ (μM)	FDPS IC_50_ (μM)	Cell Activity (μM)
prenyl [36]	38	>100	none
homoprenyl [37]	0.43	6.6	≥1
bishomoprenyl [38]	3.6	>100	≥5
geranyl/neryl (2:1) (**7**) [39]	2.2	84	≥10
geranyl (**32**) [39]	17	57	≥10
neryl (**33**) [39]	0.38	79	≥1
geranyl epoxide [39]	23	33	none
neryl epoxide [39]	17	47	none
homogeranyl/homoneryl (3:1) (**8**) [37]	0.045	28	≥0.03
homogeranyl [40]	0.17	8.2	≥0.09
homoneryl [40]	0.075	5.6	≥0.03
bishomogeranyl [38]	2.6	33	≥5
bishomoneryl [38]	1.3	30	≥0.5
farnesyl [41]	40	58	none
homofarnesyl [36]	0.82	43	≥1
geranylgeranyl [41]	120	100	none
homogeranylgeranyl [36]	56	63	≥100
